# Recurrent background mutations in *WHI2* impair proteostasis and degradation of misfolded cytosolic proteins in *Saccharomyces cerevisiae*

**DOI:** 10.1038/s41598-017-04525-8

**Published:** 2017-06-23

**Authors:** Sophie A. Comyn, Stéphane Flibotte, Thibault Mayor

**Affiliations:** 1 Genome Science and Technology Program, University of British Columbia, Vancouver, BC V6T 1Z4 Canada; 2 Department of Biochemistry and Molecular Biology, Michael Smith Laboratories, University of British Columbia, Vancouver, BC V6T 1Z4 Canada; 3 Department of Zoology, University of British Columbia, Vancouver, BC V6T 1Z4 Canada

## Abstract

Proteostasis promotes viability at both the cellular and organism levels by maintaining a functional proteome. This requires an intricate protein quality control (PQC) network that mediates protein folding by molecular chaperones and removes terminally misfolded proteins via the ubiquitin proteasome system and autophagy. How changes within the PQC network can perturb proteostasis and shift the balance between protein folding and proteolysis remain poorly understood. However, given that proteostasis is altered in a number of conditions such as cancer and ageing, it is critical that we identify the factors that mediate PQC and understand the interplay between members of the proteostatic network. In this study, we investigated the degradation of a thermally unstable cytosolic model substrate and identified a surprisingly high number of strains in the yeast knockout collection that displayed impaired turnover of the misfolded substrate. We found that this phenotype was caused by frequent background mutations in the general stress response gene *WHI2*. We linked this proteostatic defect to the lack of activity of the stress response transcription factor Msn2, potentially under conditions where the TOR pathway is active. Our results underscore how changes to the elaborate PQC network can perturb proteostasis and impair degradation of misfolded cytosolic proteins.

## Introduction

Protein homeostasis (proteostasis) is maintained by an extensive PQC network that promotes and mediates protein folding by molecular chaperones and prevents the accumulation of misfolded proteins by targeting them for degradation via the ubiquitin proteasome system or autophagy^1^. Proteostatic balance can be challenged by exposure to a range of intrinsic or extrinsic stressors which require the cell to mount an adequate response, most notably by regulating the expression of PQC network elements in a concerted manner. Inadequate management of misfolded proteins can have deleterious consequences such as aggregation, which is characteristic of some neurodegenerative diseases that include Alzheimer’s and Parkinson’s^2^.

Proteasomal degradation of misfolded cytosolic proteins is mediated by several quality control E3 ubiquitin ligases, which typically work in concert with other chaperone proteins to recognize their substrates^3, 4^. For instance, the Hsp110 Sse1, which acts as a nucleotide exchange factor, was shown to promote ubiquitination by the Ubr1 E3 ligase in yeast^5^. As well, we proposed that the Ydj1 Hsp40 co-chaperone acts as a substrate adaptor for the Rsp5 E3 ligase upon acute heat stress^6^. In other cases, chaperone proteins are also required to promote proteolysis. The Hsp40 co-chaperone Sis1, for example, is necessary for the translocation of misfolded cytosolic proteins into the nucleus where most proteasomes reside^7^. We also recently showed that the yeast prefoldin subunit Gim3 is required to promote proteolysis by preventing aggregation of cytosolic proteins misfolded due to missense mutations^8^. Therefore, although chaperone proteins primarily promote polypeptide folding and assembly, they may also play a key role in the clearance of misfolded proteins. Understandably, the relationship between the folding and degradation machineries is complex. For example, the structurally related chaperone regulatory proteins Bag1 and Bag2 promote and inhibit, respectively, the degradation of misfolded cytosolic proteins by the CHIP E3 quality control ligase^9–11^. Therefore, a major challenge is to understand how changes in the intricate PQC network can perturb proteostasis, such as shifting the balance between folding and proteolysis.

In this study, we found that degradation of a model misfolded substrate was impaired in a surprisingly high number of strains from the yeast knockout (YKO) collection, which were associated to secondary mutations in the stress response gene *WHI2*. We linked this proteostasis defect to a deficiency in the Msn2/Msn4 transcription factor response that altered the cell’s capacity to adeptly degrade misfolded cytosolic proteins in conditions where the target of rapamycin (TOR) pathway was active.

## Results

### Multiple YKO strains display impaired proteostasis

**Figure 1:**
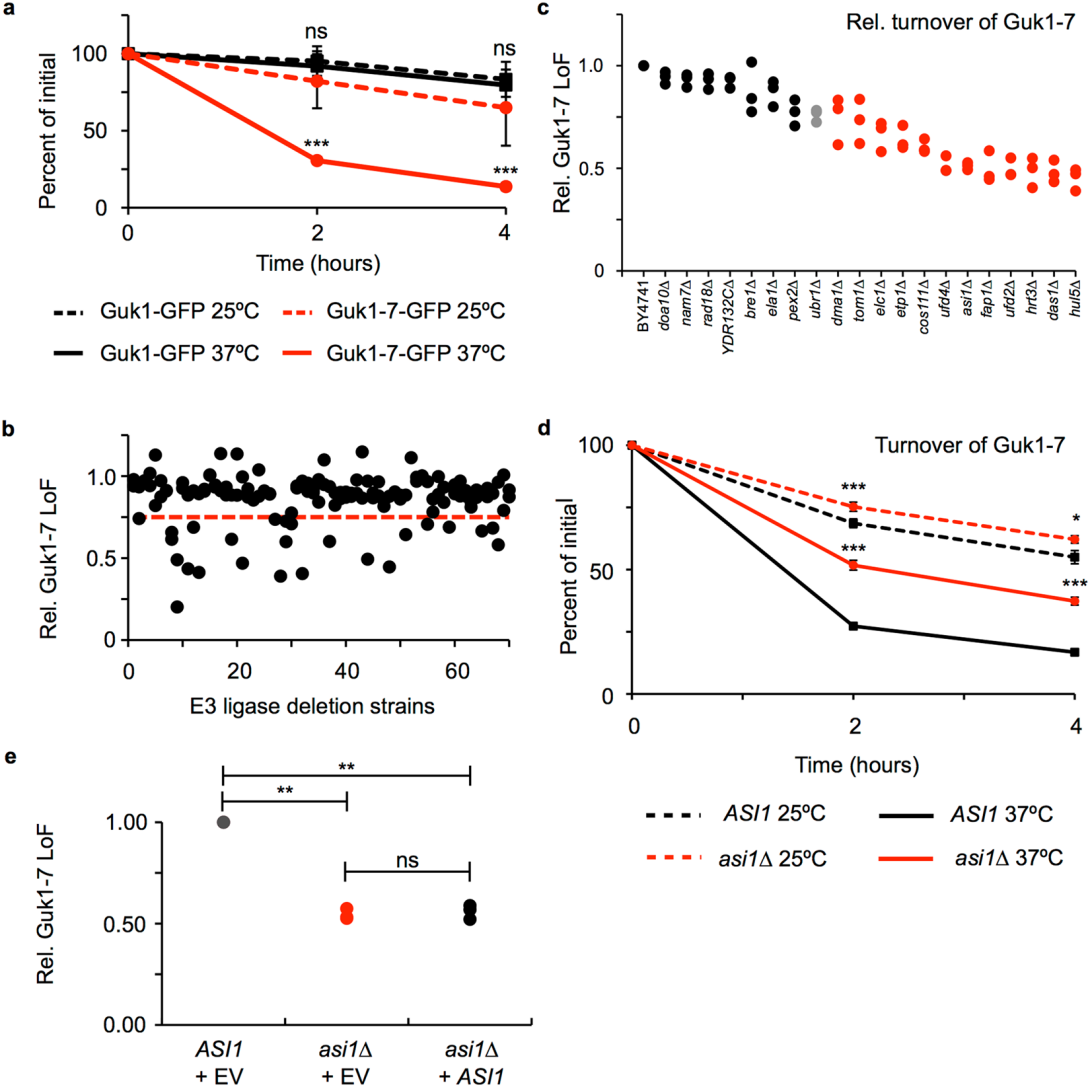
Flow cytometry based screen for E3 ligases targeting Guk1-7-GFP for degradation. (**a**) Wild type cells expressing ectopic Guk1-GFP or Guk1-7-GFP were incubated with CHX for 4 hours at 25°C or 37°C and samples were collected at the indicated time points. Results represent the mean and standard deviation of three independent experiments. (**b**) Seventy non-essential E3 ligase deletion strains expressing Guk1-7-GFP were incubated with CHX at 25°C or 37°C for 2 hours and then analyzed by flow cytometry. Red line demarks strains with a relative loss of fluorescence (Rel. LoF) value of 0.75 or lower. (**c**) The top 20 strains were selected for further validation by flow cytometry with experiments performed as in (**b**). Data points in red correspond to strains displaying Guk1-7-GFP stabilization levels higher than that of *ubr1∆*. (**d**) Wild type or *asi1∆* cells expressing Guk1-7-GFP were incubated with CHX at 25°C or 37°C for two hours prior to flow cytometry analysis. Results represent the mean and standard deviation of three independent experiments. (**e**) Wild type and *asi1∆* cells co-expressing Guk1-7-GFP and an empty vector (EV) or *ASI1* were treated as in (**d**). ns, *, ** and *** denote: not significant, p < 0.05, 0.01, and 0.005, respectively.

We previously identified the E3 ubiquitin ligase Ubr1 from a genetic screen for factors involved in degradative PQC of Guk1-7, a thermally unstable mutant allele of the guanylate kinase Guk1 that is degraded after shifting cells to 37°C (Fig. 1a)^8^. Ubr1 activity alone, however, was not sufficient to account for the bulk of substrate degradation by the proteasome^8^. To identify another E3 ubiquitin ligase responsible for the degradation of the Guk1-7-GFP model substrate, we screened a collection of 70 non-essential E3 ligase deletion strains that were individually transformed with a plasmid encoding the Guk1-7-GFP fusion under the GPD promoter. Cultures were grown at 25°C and then divided and incubated in the presence of cycloheximide (CHX) for two hours at either 25°C or 37°C before performing flow cytometry analysis. For each deletion strain, the relative difference in median GFP fluorescence intensity between samples incubated at 25°C or 37°C was normalized to that of the wild type strain in order to calculate the relative loss of fluorescence (Rel. LoF) of Guk1-7-GFP (Fig. 1b). The collection was screened twice, and strains that had a relative loss of Guk1-7-GFP fluorescence value of 0.75 or lower, in at least one of the two rounds, were selected for further validation. A total of 20 strains met this criterion and were further analysed by flow cytometry in three independent experiments (Fig. 1c). In agreement with our previous findings, deleting *UBR1* led to a 25% lower average loss of Guk1-7-GFP fluorescence compared to that of wild type cells^8^. Surprisingly, we identified twelve E3 ligase deletion strains with a greater impairment of Guk1-7-GFP degradation than that observed in *ubr1∆* cells. Of these, seven strains had an average relative loss of Guk1-7-GFP fluorescence value of 0.5 or lower. These results indicate that an unusually high number of strains from our yeast knockout collection have a reduced capacity to eliminate misfolded cytosolic proteins.

### A secondary mutation in *WHI2* is linked to impaired proteostasis

We confirmed the results of our screen by performing CHX chase experiments with cells lacking *ASI1*, a member of the nuclear inner membrane Asi ubiquitin ligase complex (Fig. 1d)^12^. To determine whether the impaired turnover of the model substrate was caused by the absence of Asi1, we co-expressed Guk1-7-GFP with *ASI1* under its endogenous promoter or with a control empty vector (EV) in *asi1∆* cells. Addition of wild type *ASI1* did not re-establish normal model substrate degradation levels in these cells (Fig. 1e). We obtained similar results with the six remaining E3 ligase deletions that displayed stabilization values comparable with *ASI1* (*DAS1*, *FAP1*, *HRT3*, *HUL5*, *UFD2*, and *UFD4*) (Supplementary Figs 1 and 2). This data implied that the decrease in Guk1-7-GFP degradation conferred by these strains could not be attributed to the absence of the assessed E3 ubiquitin ligases, but rather to that of another factor, such as a background mutation, or potentially an epigenetic factor. Similarly, we previously isolated several hits in a genome wide screen for factors involved in degradative PQC (e.g., YER071C and YJL141C) that we could not confirm after addback experiments expressing the deleted gene from a plasmid, indicating that this phenomenon was not limited to E3 ligase mutant cells^8^.

**Figure 2:**
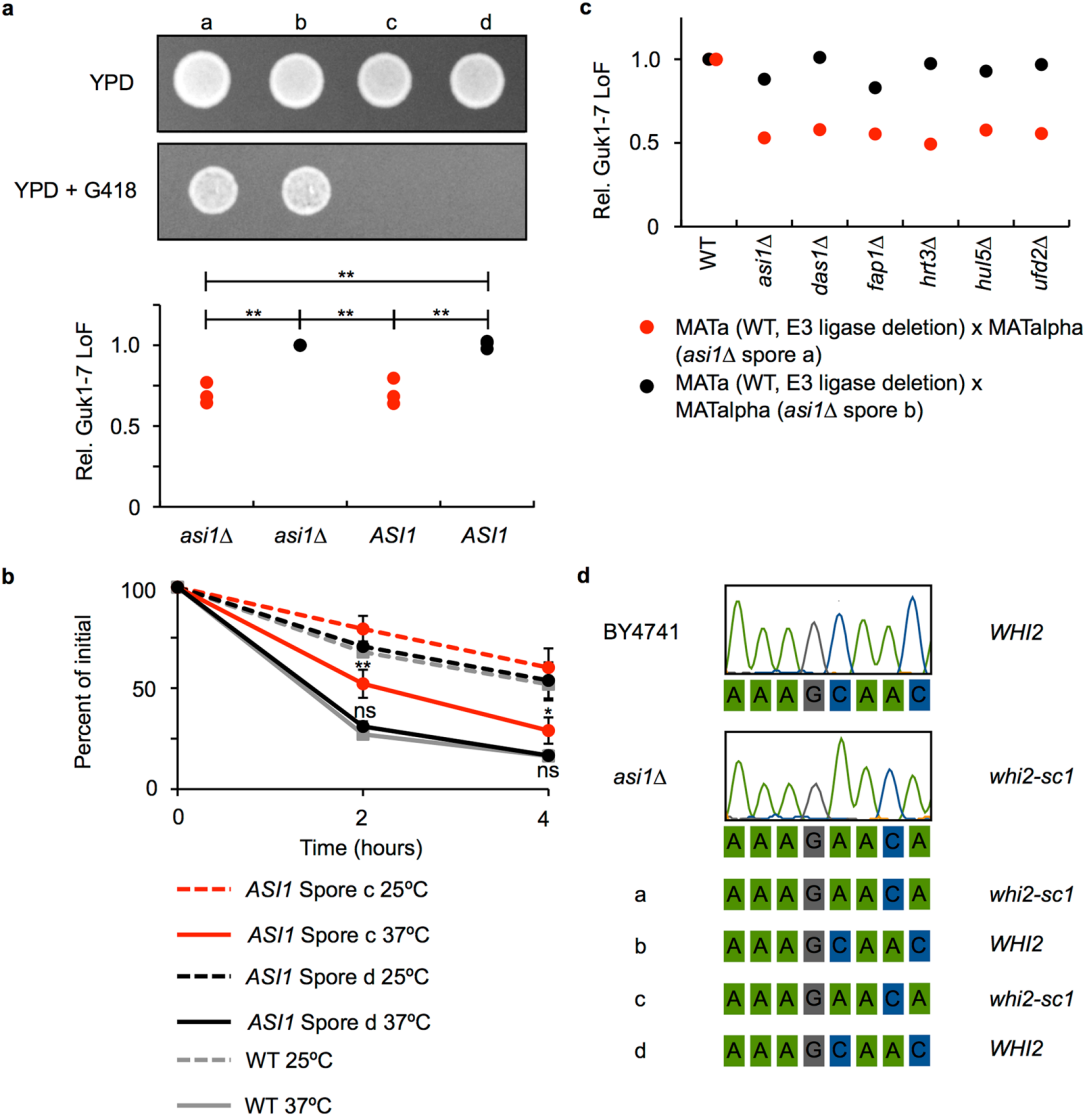
Mutations in *WHI2* segregate with the Guk1-7-GFP stability phenotype. (**a**) Analysis of a representative tetrad derived from the mating of *MAT*a *asi1∆* and *MAT*alpha BY4742 cells. Growth was assessed on the indicated plates (top) and the relative loss of fluorescence (Rel. LoF) of Guk1-7-GFP was determined following incubation with CHX at 25°C or 37°C for 2 hours (bottom). Results represent three independent experiments and p values were calculated with a one-way ANOVA and post-hoc Tukey HSD to assess significance, **denotes p < 0.01. (**b**) Spores c and d, produced from the *asi1∆* backcross shown in (**a**), expressing Guk1-7-GFP were incubated with CHX at 25°C or 37°C for four hours. Samples were analysed by flow cytometry at the indicated time points. The results represent the mean and standard deviation of three independent experiments. P values were calculated with a two-tailed unpaired Student’s *t*-test (*, **, and ns denote p < 0.05, 0.01, and not significant, respectively). (**c**) Complementation test in which the indicated diploids expressing Guk1-7-GFP were incubated with CHX for two hours at 25°C or 37°C and analysed by flow cytometry. *MAT*a E3 ligase deletion strains or WT BY4741 were mated with *MAT*alpha *asi1∆* cells from spore a (red) and b (black). (**d**) Whole genome sequencing of BY4741, *asi1∆*, and the four *asi1∆* backcross spores (shown in **a**) revealed a single base pair deletion in the coding sequence of *WHI2* that co-segregates with the Guk1-7-GFP stability phenotype.

We next sought to determine whether the observed phenotype was caused by a single background mutation. Therefore, we performed tetrad analysis on the haploid spores obtained from backcrossing the *MAT*a *asi1∆* strain to wild type *MAT*alpha BY4742 cells. No discernable difference in growth rate was seen across the dissected spores. The selection marker KanMX, which confers resistance to geneticin in *asi1∆* cells, and the impaired degradation phenotype did not appear to be linked. However, each segregated to the expected 2:2 ratio as is shown by a representative tetrad set (Fig. 2a). We obtained similar data with *das1*∆ cells (Supplementary Fig. 3). Data from CHX chase experiments confirmed that Guk1-7-GFP levels were significantly higher in spore c (*ASI1*) compared to those in the parental wild type and spore d cells after a two hour (p = 0.001 and p = 0.002) and four hour (p = 0.025 and p = 0.028) incubation at 37°C (Fig. 2b). These experiments indicate that the stabilization phenotype in the *asi1∆* strain was likely due to background mutations at a single locus.

Next, we performed a complementation test to determine whether the secondary mutations responsible for the stability phenotype observed in the seven E3 ligase deletion strains are in the same locus, or different loci. Heterozygous diploids were produced by mating either a wild type strain (BY4741) or each of the seven E3 ligase deletions to two haploid strains derived from the *asi1∆* backcross: one that presumably contained the secondary mutation (spore a in Fig. 2a), and the other that did not (spore b). Turnover of Guk1-7-GFP was normal in heterozygous diploids produced from mating spore a and BY4741 and diploids produced from crossing spore b with any of the E3 deletion mutants, or the wild type BY4741 (Fig. 2c). Conversely, all heterozygous diploids derived from mating E3 ligase deletions with spore a (harbouring the secondary mutation) demonstrated increased Guk1-7-GFP stability. These results indicate that these background mutations belong to the same complementation group, and suggest that the secondary mutations present in each strain are in the same gene.

To identify the locus containing the secondary mutation, we performed whole-genome sequencing on four haploid spores and their parental wild type and *asi1∆* strains. Secondary mutations in the genes *GLO4* and *WHI2*, which are approximately 3,000 bp apart on chromosome fifteen, co-segregated with the strains harbouring the increased Guk1-7-GFP stability phenotype. Interestingly, secondary mutations in the general stress response gene *WHI2* have been identified previously in yeast knockout collections and genome evolution studies^13–16^. We identified a single nucleotide deletion in the coding sequence of *WHI2*. This mutation, hereinafter referred to as *whi2-sc1*, produces a frameshift introducing a premature stop codon and likely results in a loss of *WHI2* function (Fig. 2d). By contrast, the coding sequence of *GLO4*, a mitochondrial glyoxalase, contained a single missense mutation.

### Proteostasis is impaired owing to secondary mutations in *WHI2*

**Figure 3:**
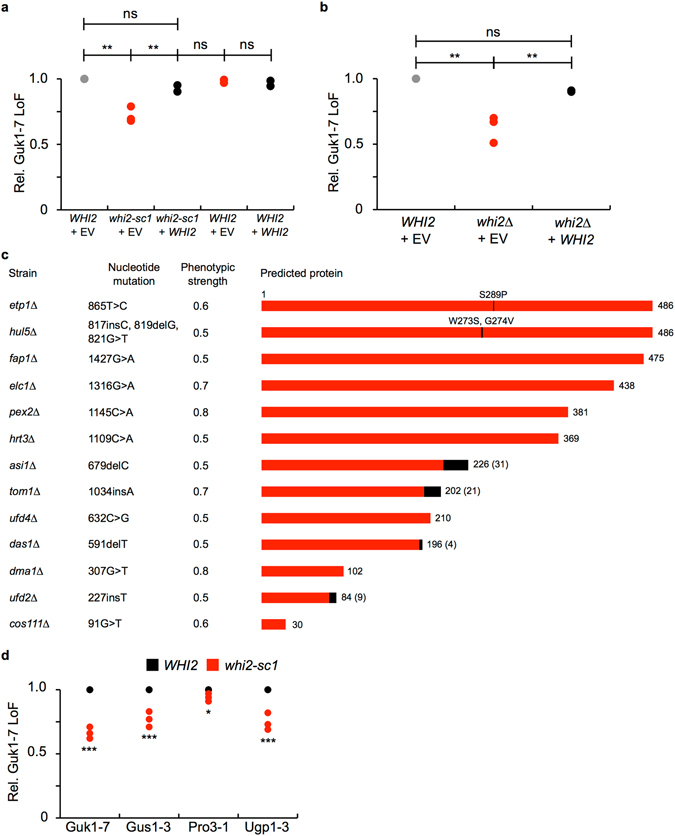
Absence of *WHI2* leads to Guk1-7-GFP stability. (**a**) Relative loss of fluorescence (Rel. LoF) of Guk1-7-GFP in cells from spores c and d that carried a control empty vector (EV) or *WHI2* were incubated with CHX at 25°C or 37°C for two hours and then analysed by flow cytometry. Results represent three independent experiments and p values were calculated with a one-way ANOVA and post-hoc Tukey HSD to assess significance (ns and ** denote not significant and p < 0.01, respectively). (**b**) Wild type and *whi2∆* cells expressing Guk1-7-GFP along with an empty vector (EV) control or *WHI2* were treated and analysed as in (**a**). (**c**) Positions of the *WHI2* mutations in the indicated strains together with the predicted protein lengths depicted in red. Black boxes denote the C-terminal mismatch extensions. (**d**) Relative turnover of the indicated mutated proteins in *WHI2* and *whi2-sc1* cells that were treated and analyzed as in (**a**). Results represent three independent experiments. P values were calculated with a two-tailed unpaired Student’s *t*-test (** and *** denote p < 0.01 and 0.005, respectively).

To determine whether the mutation in *WHI2* caused the observed stabilization, we expressed the *WHI2* wild type open reading frame (ORF) from a plasmid in cells derived from the backcross. Whereas the addition of an empty vector did not rescue the phenotype, addition of *WHI2* re-established normal Guk1-7-GFP degradation levels (Fig. 3a). Likewise, degradation of Guk1-7-GFP was impaired in the null *whi2∆* strain from the yeast knockout collection, and degradation could be restored by the expression of *WHI2* (Fig. 3b). Intriguingly, we found that *glo4∆* cells had a similar reduction in Guk1-7-GFP degradation. Subsequent sequencing of the *WHI2* locus in *glo4∆* cells identified two point mutations that produce a premature stop codon, and expression of *WHI2* in *glo4∆* cells re-established normal turnover of Guk1-7-GFP (Supplementary Fig. 3). These results suggest that the effect observed in *glo4∆* cells is attributed to a loss of *WHI2* function, not of *GLO4*, and that loss of *WHI2* function is sufficient to strongly impair degradation of a misfolded cytosolic model substrate.

We next sought to confirm that *WHI2* was also mutated in the other E3 ligase deletion strains in which Guk1-7-GFP degradation was impaired. Mutations in *WHI2* sensitize cells to exposure to acetic acid, which lends itself to a convenient assay for Whi2 function^13^. The *whi2∆* and all seven E3 ligase deletion strains were sensitive to acetic acid treatment (Supplementary Fig. 3). Expressing *WHI2* from a plasmid under its endogenous promoter restored cell viability in all strains, confirming data from the complementation test, suggesting that all strains contain secondary mutations in the same locus (Fig. 2c). We proceeded to sequence the *WHI2* gene, including approximately one hundred base pairs upstream and downstream of the start and stop codons, in all twenty of the top E3 ligase deletion strains from our screen. Whereas *ubr1∆* and six other strains had no apparent mutations, we identified unique *WHI2* mutations in a total of thirteen strains (Fig. 3c). Of the *WHI2* mutations identified, eleven are predicted to produce truncated proteins resulting from the introduction of a premature stop codon. Four of the eleven also contain additional C-terminal extensions (ranging from 4 to 31 amino acids in length) as the result of frameshift mutations. The mutations identified are relatively evenly dispersed along the length of the protein with the exception of a mutation free region, seventy amino acids in length, which was found approximately three quarters of the way into the protein. The presence of *WHI2* mutations is unlikely to be restricted to E3 ligase mutant cells. Indeed, three other studies have reported the presence of secondary mutations in the *WHI2* locus of strains from the yeast deletion collection^14, 16, 17^. Mutations in *WHI2* have been speculated to provide a favourable fitness advantage under certain environments or in combination with other compensatory mutations^15, 18^. However, we did not observe differences in the growth of haploid cells following tetrad analysis.

We next determined whether the degradation of other misfolded cytosolic proteins was impaired due to a loss of *WHI2* function. We assessed the levels of three other GFP fusion mutants: Gus1-3, Pro3-1, and Ugp1-3 that are encoded by thermosensitive alleles of glutamyl-tRNA synthetase, delta-1-pyrroline-5-carboxylate reductase, and UDP-glucose pyrophosphorylase, respectively. We previously demonstrated that these mutant cytosolic proteins are degraded upon shifting cells to a higher temperature^8, 19^. As for the Guk1-7 mutant, degradation of all three alleles was impaired in *whi2-sc1* cells, although Pro3-1 to a lesser extent than the others (Fig. 3d). Altogether, these results indicate that loss of *WHI2* function has a varied impact on the degradation of misfolded cytosolic proteins and that some quality control substrates are more dependent on the presence of Whi2.

### Mutant *WHI2* reduces substrate ubiquitination

**Figure 4:**
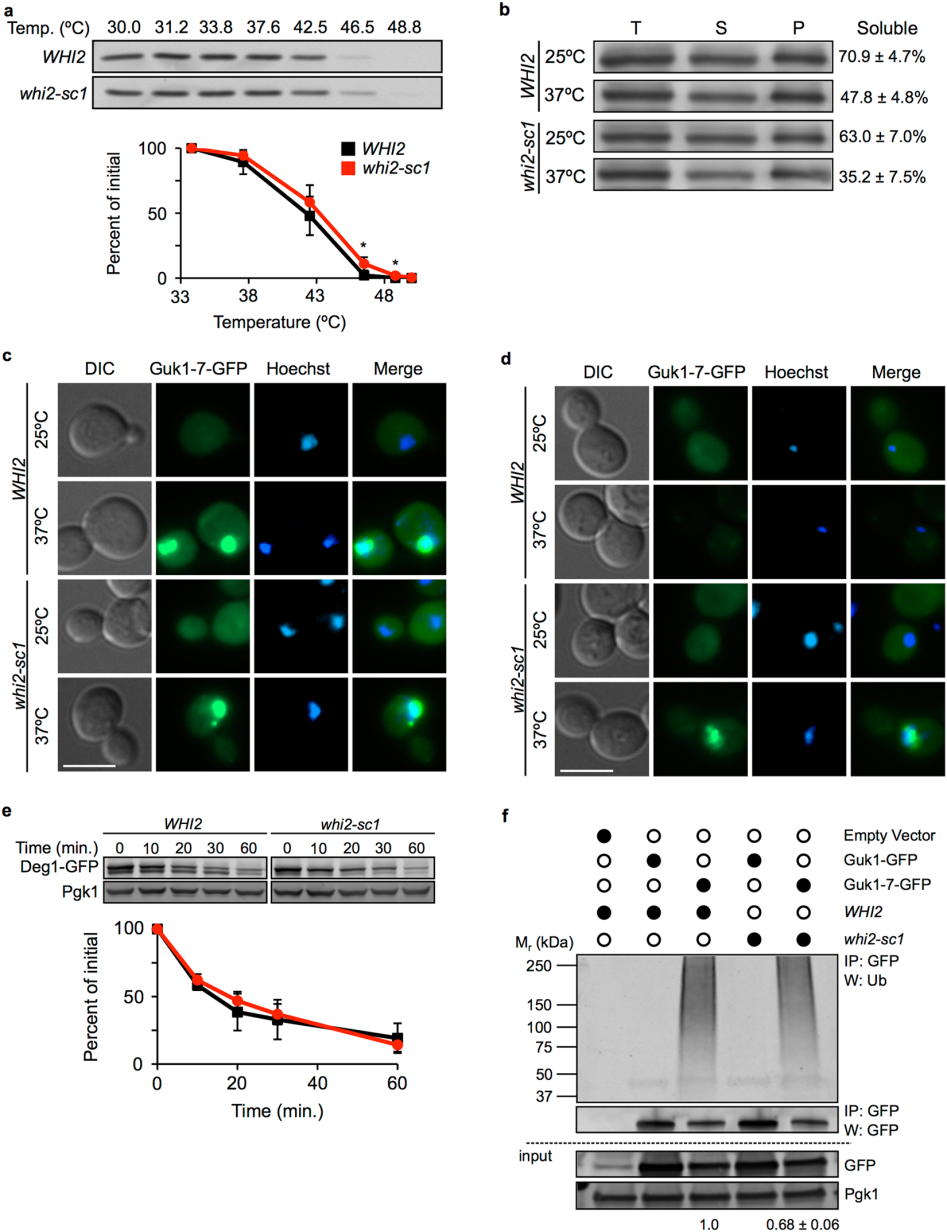
*whi2∆* promotes Guk1-7-GFP stability through reduced ubiquitination. (**a**) Cellular thermal shift assay of Guk1-7 fused to a six histidine tag in lysates of *WHI2* and *whi2-sc1* cells grown at 25°C. One representative anti-His western blot is shown. The graph represents the means and standard deviations of Guk1-7 levels from three independent experiments. P values were calculated with a two-tailed unpaired Student’s *t*-test (*denotes p < 0.05). (**b**) *WHI2* and *whi2-sc1* cells expressing Guk1-7-GFP were grown at 25°C or shifted to 37°C for 20 min. Total cell lysate (T), soluble (S), and pellet fractions (P) were immunoblotted with an anti-GFP antibody. One representative blot is shown and the ratio of soluble fraction to total cell lysate is noted and represents the mean and standard deviation of three independent experiments. Although, slightly lower levels were recovered in the soluble fractions of *whi2-sc1* cells, the differences with wild type cells were not significant. (**c**) *WHI2* or *whi2-sc1* cells expressing Guk1-7-GFP were grown at 25°C and then incubated at 25°C or 37°C for 20 minutes with CHX before fixation and imaging. The scale bar represents 5 µm. (**d**) As in (**c**) except cells were incubated in the presence of CHX for 2 hours at 25°C or 37°C. (**e**) Turnover of a constitutive substrate of the UPS. *WHI2* and *whi2-sc1* cells expressing Deg1-GFP under the Cup1 promoter were incubated with CHX at 30°C and samples were collected at the indicated time points. Membranes were immunoblotted with an anti-GFP antibody and an anti-Pgk1 antibody as a loading control. The graph represents the mean and standard deviation of three independent experiments. (**f**) Guk1-GFP or Guk1-7-GFP were immunoprecipitated with GFP-Trap beads from lysates of *WHI2* or *whi2-sc1* cells grown at 25°C and expressing a control empty vector, Guk1-GFP, or Guk1-7-GFP. Samples were eluted and immunoblotted with anti-ubiquitin, anti-GFP, and anti-Pgk1 antibodies. Ubiquitination levels were normalized to levels of unconjugated Guk1-7-GFP that was immunoprecipitated and averaged from three independent experiments. Values denote averaged relative quantification levels plus or minus standard deviations. Uncropped images for all Western blots can be found in Supplementary Fig. 5.

To determine how an absence of *WHI2* results in increased Guk1-7-GFP stabilization, we first needed to clarify what aspect of PQC is altered in the mutants. We first compared the thermodynamic stability of ectopically expressed Guk1-7 in cellular lysates by a cellular thermal shift assay (CETSA)^20^. Briefly, aliquots of cell lysate were heated to different temperatures, cooled, and then centrifuged to separate soluble fractions from precipitated proteins. Our protein of interest, Guk1-7, could then be detected in the soluble fraction by western blotting to generate thermal melt curves. The presence of Guk1-7 in the soluble fraction decreased rapidly at temperatures above 42°C in extracts from both wild type and *whi2-sc1* strains (Fig. 4a). While not marked, slightly more Guk1-7 remained in the soluble fraction of *whi2-sc1* lysates compared to wild type at 46.5°C and 48.8°C (p = 0.04 and p = 0.032). We next asked whether an absence of Whi2 influences protein folding by assessing NP-40 solubility. There was no significant difference in Guk1-7-GFP solubility between wild type and *whi2-sc1* cells for cultures grown at 25°C, or following a short twenty minute incubation at 37°C (p = 0.18 and p = 0.07) (Fig. 4b). We further examined Guk1-7-GFP by fluorescence microscopy. After a twenty minute incubation at 37°C in the presence of CHX, Guk1-7-GFP was sequestered into inclusions that appear adjacent to, or overlapping with, nuclear staining in both wild type and *whi2-sc1* cells (Fig. 4c). Consistent with our flow cytometry data (Fig. 1d), following a two hour incubation at 37°C in the presence of CHX, Guk1-7-GFP accumulated in large inclusions in *whi2-sc1* cells (63% of cells contained an average of 1.2 inclusions, n = 72) whereas less Guk1-7-GFP was visible in *WHI2* cells, which contained smaller inclusions (48% of cells contained an average of 1.5 inclusions, n = 67) (Fig. 4d). Together, these data suggest that an absence of *WHI2* leads to slower clearance of Guk1-7-GFP without increasing thermal stability or promoting substrate solubility.

It is possible that mutations in *WHI2* might generally alter the ubiquitin proteasome system. However, using the known proteasome substrate Deg1-GFP, we found no significant difference in its degradation in *whi2-sc1* cells compared to wild type cells (p = 0.31, 0.40, 0.70, 0.52 for 10, 20, 30, and 60 minute time points, respectively) (Fig. 4e). We next asked whether an absence of *WHI2* could affect Guk1-7-GFP ubiquitination. Quantification of ubiquitin levels was performed in three independent experiments and measured following pulldown of Guk1-GFP and Guk1-7-GFP from cultures grown at 25°C. Normalizing the ubiquitin levels to the amount of GFP tagged substrate eluted revealed that the poly-ubiquitination signal of Guk1-7-GFP in *whi2-sc1* cells was significantly reduced compared to wild type cells (Fig. 4f; average 32% reduction ± 6%, p = 0.0008, n = 3). Together, these data suggest that mutated *WHI2* could impair Guk1-7-GFP degradation, in part by decreasing substrate ubiquitination.

### Reduced proteostasic capacity in *WHI2* mutants is linked to Msn2

**Figure 5:**
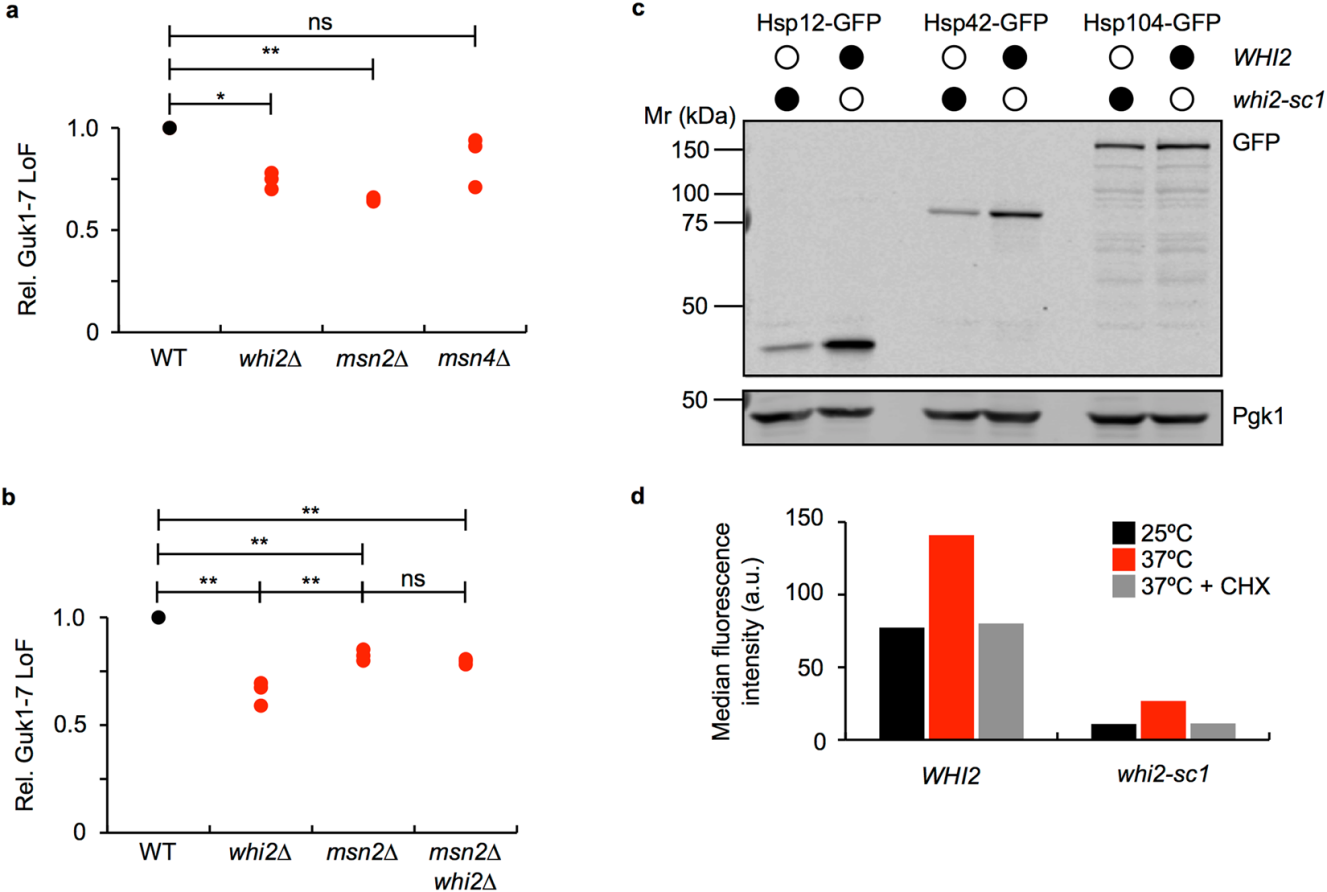
Msn2 is linked to reduced proteostatic capacity in *WHI2* mutants. (**a**) Wild type, *whi2∆*, *msn2∆*, and *msn4∆* cells expressing Guk1-7-GFP were analysed by flow cytometry following a two hour incubation at 25°C or 37°C in the presence of CHX. P values were calculated with a one-way ANOVA and post-hoc Tukey HSD to assess significance (*, **, and ns denote p < 0.05, 0.01, and not significant, respectively). (**b**) Wild type, *whi2∆*, *msn2∆*, and *msn2∆whi2∆* cells expressing Guk1-7-GFP were analysed by flow cytometry following a two hour incubation at 25°C or 37°C in the presence of CHX. P values were calculated with a one-way ANOVA and post-hoc Tukey HSD to assess significance (** and ns denote p < 0.01 and not significant, respectively). (**c**) *WHI2* and *whi2-sc1* strains with either Hsp12, Hsp42, or Hsp104 endogenously tagged with GFP were grown at 25°C. Membranes were immunoblotted with an anti-GFP antibody and an anti-Pgk1 antibody as a loading control. (**d**) *WHI2* and *whi2-sc1* strains with Hsp12 endogenously tagged with GFP were grown to log phase and then incubated at 25°C, 37°C, or 37°C in the presence of CHX for 2 hours prior to anlysis by flow cytometry. Uncropped images for all Western blots can be found in Supplementary Fig. 5.

Exposure to stressors such as heat, oxidative or osmotic shock, and nutrient starvation results in the transcriptional activation of approximately 200 genes in yeast^21^. Activation of this general stress response is mediated by binding of the partially redundant zinc finger transcription factors Msn2 and Msn4 to stress response elements (STRE) in the promoters of stress-response genes^21, 22^. Studying the general stress response, mediated by Msn2/4 signalling, has gained renewed importance in light of a recent study showing that the majority of genes induced by heat shock in yeast are activated through Msn2/4 activity and not by Hsf1, as was previously believed^21^. Msn2 has been shown to form a complex with the plasma membrane phosphatase Psr1 and Whi2^23^. Therefore, we wished to assess the stability of Guk1-7-GFP in *msn2∆* cells to determine whether the mutant *WHI2* phenotype was potentially the result of an altered general stress response. Notably, we observed that an absence of *MSN2*, but not *MSN4*, led to a significant increase in Guk1-7-GFP compared to wild type (p = 0.003) with levels similar to those seen in *whi2∆* cells (Fig. 5a). We confirmed that *WHI2* was not mutated in *msn2∆* cells. We then examined Guk1-7-GFP stability in the double *msn2∆whi2∆* mutant and observed no additive effect (Fig. 5b). These results indicate that the reduced turnover in *whi2∆* cells and in *msn2∆* cells was likely caused by the disruption of the same pathway Therefore, the decreased Guk1-7-GFP degradation in the absence of Whi2 is likely associated to a general impairment of stress response factors acting downstream of Msn2. To confirm that mutants of *WHI2* affect the Msn2 transcription factor, we assessed whether protein levels of Msn2 responsive genes were altered in the absence of Whi2 function. Therefore, we endogenously tagged Hsp12, Hsp42, and Hsp104 with GFP in wild type and *whi2-sc1* strains, as these three heat shock proteins have been shown to be targets of Msn2 and their expression is induced following heat shock^24^. Steady state protein levels of Hsp12 and Hsp42 were approximately 90% lower in *whi2-sc1* cells compared to wild type when examined by western blot (Fig. 5c). Levels of Hsp104 appear to be less affected by a lack of Whi2 function. Flow cytometry experiments support the finding of lower steady state levels of Hsp12 (Fig. 5c) at 25°C in *whi2-sc1* cells compared to wild type. Hsp42 and Hsp104 showed a similar trend, but their levels were much lower in both wild type and *whi2-sc1* cells (Supplementary Fig. 4). In wild type cells, levels of GFP fluorescence increased for all three Hsp proteins following incubation at 37°C (but not in the presence of CHX), whereas GFP fluorescence levels were lower in *whi2-sc1* cells (Fig. 5d and Supplementary Fig. 4). These data suggest that the steady state levels of the assessed Msn2 stress responsive genes are altered in *whi2-sc1* cells, even in the absence of stress.

**Figure 6:**
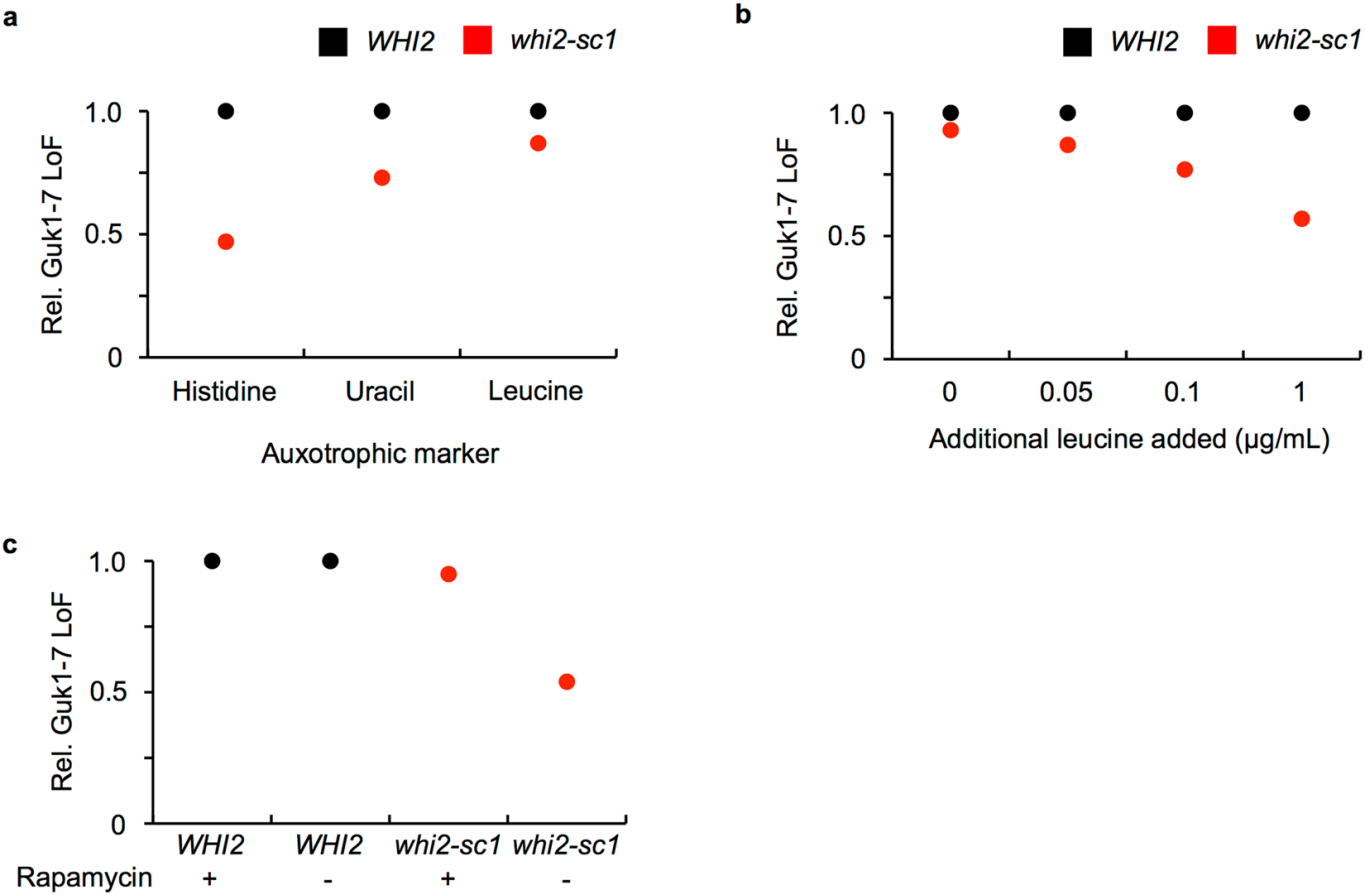
Deficient proteostatic response under conditions where the TOR pathway is active. (**a**) Guk1-7-GFP was expressed from CEN/ARS plasmids with histidine, uracil, or leucine auxotrophic markers in wild type or *whi2-sc1* cells. Cultures were incubated with CHX for two hours at 25°C or 37°C before being analysed by flow cytometry. (**b**) Wild type and *whi2-sc1* cells were co-transformed with Guk1-7-GFP and pRS315 (*LEU2*). Cultures were grown in synthetic drop out media containing the indicated amounts of leucine and then incubated for two hours in the presence of CHX at 25°C or 37°C before flow cytometry analysis. (**c**) *WHI2* and *whi2-sc1* cells were handled as in (**a**) except for being treated with 200 nM rapamcyin for two hours at 25°C prior to the two hour incubation at 25°C or 37°C.

The activity of the Msn2/Msn4 transcription factors is regulated and fine tuned by multiple factors including the glucose-responsive cyclic AMP (cAMP)-protein kinase A^25^. Intriguingly, while performing *WHI2* addback experiments we noticed a pronounced decrease in Guk1-7-GFP stability when adding a second plasmid bearing the auxotrophic marker leucine. To further investigate this observation, we transformed wild type and *whi2-sc1* cells with a plasmid expressing Guk1-7-GFP that contained one of the following selection markers: histidine, uracil, or leucine. Consistent with our previous data, the loss of Guk1-7-GFP fluorescence was 53% lower in *whi2-sc1* cells compared to wild type when the histidine marker was used (Fig. 6a). However, when *whi2-sc1* cells containing the leucine selection marker were grown in synthetic media without additional leucine, Guk1-7-GFP degradation persisted and levels were approximately two fold lower. Uracil selection resulted in an intermediate phenotype. Leucine was previously shown to activate the TORC1 kinase complex that can also inhibit Msn2/4^26–29^. TOR is a conserved serine threonine protein kinase which, as part of the TORC1 complex, promotes growth by linking protein synthesis to extrinsic signals such as nutrient levels and environmental stresses. Treating cells with the antifungal rapamycin mimics nutrient starvation and stress, thereby inhibiting TORC1 and allowing a number of stress and growth responsive transcription factors (such as Msn2) to translocate into the nucleus^30^. One possibility is that Whi2 is only required to maintain Msn2 active when TORC1 is stimulated in the presence of high levels of exogenous leucine. In support of this view, impaired Guk1-7-GFP degradation was restored in *whi2-sc1* cells upon the addition of increasing amounts of leucine (Fig. 6b). Conversely, when TORC1 was inhibited in the presence of rapamycin, Guk1-7-GFP degradation in *whi2-sc1* cells was comparable to that of wild type (Fig. 6c). These data would suggest that Whi2 is required for Msn2 function under conditions where TOR is active.

## Discussion

Temperature sensitive alleles have proven to be fruitful model substrates when used to elucidate the existence and function of PQC pathways. In this study, we used the thermally unstable Guk1-7 allele as a model PQC substrate to identify the E3 ubiquitin ligase, or ligases, responsible for it’s temperature dependent degradation. Screening a yeast deletion collection using a flow cytometry based approach resulted in the identification of a number of putative E3 ligase hits. Further validation, however, suggested that the phenotype observed was produced by an indirect effect. Subsequent complementation analysis and whole genome sequencing revealed secondary mutations in the general stress response factor *WHI2* that account for the observed increase in Guk1-7-GFP stability. *WHI2* promotes Guk1-7-GFP degradation via substrate ubiquitination with no effect on solubility or thermal stability.

We previously identified the E3 ubiquitin ligase Ubr1 from a genetic screen for factors involved in degradative protein PQC of Guk1-7^8^. Ubr1 activity alone, however, was not sufficient to account for the bulk of substrate degradation. In some cases, the nuclear E3 ligase San1 and cytosolic ligase Ubr1 have been shown to act in parallel to degrade cytosolic substrates^5, 19^. We found no role for San1, and no additive effect with the double *ubr1∆san1∆* mutant in the degradation of the Guk1-7 substrate. Therefore, in this study we wanted to identify the E3 ubiquitin ligases, in addition to Ubr1, that are responsible for the proteasomal degradation of Guk1-7. We identified a number of potential hits in our screen which included: *ASI1*, *DAS1*, *FAP1*, *HRT3*, *HUL5*, *UFD2*, and *UFD4*. *ASI1* is a RING domain family member localized to the inner nuclear membrane and is part of the Asi complex that acts as a branch of the ERAD degradation pathway independent from *HRD3* and *DOA10* ^31, 32^. Both *DAS1* and *HRT3* are putative F-box SCF ubiquitin ligases^33, 34^. A homologue of the human transcription factor NF-X1, *FAP1* also confers resistance to rapamycin by acting as a ligand for FKBP12^35^. *HUL5* is a member of the HECT ubiquitin ligase family. Involved in cytoplasmic PQC of short-lived misfolded proteins, it is also necessary for the increased ubiquitination observed as part of the heat shock quality control response^36^. *UFD2* is both an E3 and E4 enzyme with mutants being hypersensitive to protein misfolding stressors^37^. Finally, *UFD4*, like *HUL5*, is also a member of the HECT family of E3 ligases and physically interacts with Ubr1 to increase processivity of ubiquitin chain formation in the N-end rule pathway^38^. While an absence of these seven genes resulted in significant stabilization of our model substrate, we were surprised to have identified some of them in our screen. Guk1 is found in both the cytoplasmic and nuclear compartments of yeast cells as assessed by GFP tagging and fluorescence microscopy^8, 39^. It was, therefore, not entirely unexpected for some hits to be nuclear proteins. However, based on the ascribed functions for some of the ligases, and the nature of our substrate, it was puzzling to have identified *ASI1*, *DAS1*, *FAP1*, and *HRT3* in our screen.

Once our seven top hits failed to validate in addback experiments, we then went on to identify secondary mutations in the coding sequence of the general stress response gene *WHI2* in thirteen of twenty E3 ligase deletion strains tested. A null *WHI2* deletion phenocopied the mutants, therefore suggesting that these mutations lead to a loss of Whi2 function. Mutations in *WHI2* have been reported in laboratory based evolution studies and are speculated to provide a favorable fitness advantage under certain environments or in combination with other compensatory mutations^15, 18^. However, we did not observe striking differences when comparing growth of haploid cells on YPD following tetrad analysis. To our knowledge, ours is the first report of *WHI2* mutations in E3 ubiquitin ligase deletion strains. Nevertheless, the presence of *WHI2* mutations is unlikely restricted to E3 ligase mutant cells, as we have demonstrated here. Three other studies have reported the presence of secondary mutations in the *WHI2* locus of strains from the yeast deletion collection^14, 16, 17^. These reports highlight the confounding effects of suppressor mutations on genetic studies performed using yeast deletion collections. In the most recent study, approximately 30% of all strains sequenced carried unique mutations in *WHI2* and/or five other genes^16^. Most of the mutations identified were frameshift or nonsense mutations, suggesting a loss of function phenotype as we have shown in this report. Finally, it was found that serially passaging *whi2∆* strains under conditions with a prolonged stationary phase resulted in an increased abundance of the deletion strains relative to a wild type control. This suggests that secondary mutations in these genes might be found at higher frequencies as the result of selecting for mutants that delay the onset of the stationary phase under laboratory conditions, such as those used to create the yeast deletion collection.

As part of the general stress response, Whi2 forms a complex with the plasma membrane phosphatase Psr1 and the zinc finger transcription factor Msn2^23^. Upon exposure to stress, Msn2 translocates into the nucleus where it can bind to stress response elements (STRE) in promoters of stress responsive genes. We found that like *WHI2*, an absence of *MSN2* had a positive effect on Guk1-7-GFP stability. We also demonstrated that some Msn2 stress responsive genes have lower steady state levels in strains lacking Whi2 function. This suggests that the increase in Guk1-7-GFP stability observed in *WHI2* mutants is mediated by factors downstream of Msn2. Alternatively, *WHI2* may be responsible for Msn2 stability and an absence of, or mutations in, *WHI2* could result in lower levels of Msn2 and therefore an altered general stress response in these strains. Studying the general stress response, mediated by Msn2/4 signalling, has gained renewed importance in light of a recent study examining the role of Hsf1 in the transcriptional response to heat shock. The authors of that study concluded that the majority of genes induced by heat shock in yeast are activated through Msn2/4 activity and not by Hsf1, as was previously believed^21^.

While performing *WHI2* addback experiments, we observed that Guk1-7-GFP stability depended upon adequate levels of leucine being present in the media. Amino acids, especially leucine, have been shown to regulate the TOR signalling pathway^29^. TOR is a conserved serine threonine protein kinase, which as part of the TORC1 complex promotes growth by linking protein synthesis to extrinsic signals such as nutrient levels and environmental stresses. Treating cells with the antifungal rapamycin mimics nutrient starvation and stress, thereby inhibiting TORC1 and allowing a number of stress and growth response transcription factors (such as Msn2) to translocate into the nucleus^30^. We propose a model in which Whi2 is required to maintain Msn2 functionality when TORC1 is active (*e.g*., in the presence of high concentrations of leucine in the media). Under these conditions, mutations in *WHI2* would result in Msn2 phosphorylation and inhibition of the downstream induction of stress responsive genes, thereby preventing degradation of misfolded cytosolic proteins such as Guk1-7. Conversely, when TORC1 activity is decreased or inhibited (*e.g*., in *LEU2* cells deprived of exogenous leucine or in the presence of rapamycin) Msn2 is activated in a *WHI2* independent manner and Guk1-7 is degraded with similar dynamics as in wild type cells. Our findings of a link between TORC1 and the Whi2 general stress response in the degradation of our model substrate might help explain some puzzling results we obtained from a previous screen we conducted with Guk1-7-GFP^8^. In our previous screen we had a high false positive rate and a number of hits which, at the time, were unexpected. Two such hits were the kinases Yak1 and Rim15, which have been shown to directly phosphorylate Msn2 *in vitro* and to translocate from the cytosol into the nucleus following mTORC1 inhibition^40, 41^. There is one striking point that remains unanswered: Msn2 is thought to be mostly active after a stress to induce expression of stress response genes, but in our conditions translation of newly expressed genes would be prevented by cycloheximide. Therefore, the impaired turnover of our model substrate is likely the result of an imbalance of the proteostatic network prior to the stress (*i.e*., Msn2 basal activity is also required in unstressed conditions). Indeed, protein steady state levels of several Msn2 targets (e.g. Hsp12) were lower in *whi2-sc1* cells.

## Materials and Methods

### Yeast strains, media, and growth conditions

The *Saccharomyces cerevisiae* strains used in this study are listed in Table S1. Yeast strains were cultured in synthetic media with 2% dextrose (lacking the appropriate amino acids for plasmid selection) or YPD (1% yeast extract, 2% peptone, 2% dextrose) and grown at 25°C with shaking unless indicated otherwise. When not specified otherwise, cultures in log phase were obtained by diluting overnight saturated cultures grown at 25°C to an OD_600_ = 0.2 and grown for 4–6 hours until log phase OD_600_ = 0.8–1.0 was reached.

### Plasmids

Plasmids used in this study are listed in Table S3. Guk1-GFP (BPM453), Guk1-7-GFP (BPM458), and Guk1-7-His_6_ (BPM717) expressed from the *GPD1* promoter in pRS313 were generated in a previous study^8^; Guk1-7-GFP was subcloned with ApaI and SacI sites in pRS315 to generate BPM609 and with XhoI and SacII sites in pRS316 to generate BPM781. To generate the E3 ligase addback plasmids (BPM748, *ASI1*; BPM749, *DAS1*; BPM750, *FAP1*; BPM751, *HRT3*; BPM752, *HUL5*; BPM753, *UFD2*; BPM754, *UFD4*), the open reading frames and approximately 500 bp of both endogenous 5′ and 3′ UTR was PCR amplified from genomic DNA (BY4741) and inserted into pRS316 using XhoI and XmaI sites for all but *HUL5* where SacII and XhoI sites were used. The *WHI2* (BPM863, BPM914) addback plasmids were generated as for the E3 ligases except ligated into pRS315 or pRS316 using SacI and XmaI sites, respectively.

### Flow cytometry

Cells in log phase were treated with 100 µg/mL cycloheximide and incubated at either 25°C or 37°C as indicated. GFP fluorescence was measured for 50,000 cells using a FACSCalibur (BD) flow cytometer. Median GFP fluorescence values were obtained using FlowJo software. For chase experiments, percentage remaining values were calculated by normalizing the median GFP fluorescence intensity values for each time point to the initial t = 0 measurement. To calculate the relative loss of fluorescence for single time-point measurements, the difference of GFP fluorescence values for samples incubated at 25°C or 37°C was normalized to that of the 25°C sample. To perform multiple strain comparisons, the relative loss of fluorescence values (as calculated above) for each strain was normalized to that of the wild type BY4741 strain.

### Sequencing

Whole-genome sequencing and library preparation was performed at the NextGen Sequencing facility at the University of British Columbia’s Biodiversity Research Centre. Yeast cells were grown overnight to saturation in YPD at 25°C and genomic DNA was extracted using standard protocols^42^. Barcoded libraries for each strain were created according to Illumina protocols (Illumina 2011, all rights reserved) and 100-bp paired end fragments were sequenced by pooling all six libraries and run on a single lane of an Illumina HiSeq2000. The short-read aligner BWA was used to map sequence reads to the yeast reference genome S288C version R64 (Saccharomyces Genome Database, SDG, http://www.yeastgenome.org)^43^. Single-nucleotide variants (SNVs) were identified using the SAMtools toolbox and then each SNV was annotated with a custom-made Perl script using gene data downloaded from SDG on January 21, 2014^44^. IGV viewer was used to visually inspect read alignments in the regions of candidate SNVs^45, 46^.

### *WHI2* plate assay

Yeast cultures were grown overnight at 25°C in 5 mL YPD to OD_600_ = 1–2 and then diluted to OD_600_ = 0.2 in 5 mL YPD and left to grow for 2 hours at 25°C. 1 mL was kept as an untreated control and the remaining 4 mL of culture was treated with 200 mM acetic acid for 4 hours at 25°C. Treated and untreated cultures were serially diluted fivefold in 1X PBS and plated on solid media. Plates were incubated at 30°C for 2 days before being imaged with a Gel Doc XR^+^ System (Bio-Rad).

### Cellular thermal shift assay (CETSA)

Cells expressing Guk1-7-His_6_ were grown to log phase and then lysed with glass beads in 200 µL native lysis buffer (20 mM HEPES, pH 7.5, 0.5% NP-40, 200 mM NaCl, 1X protease inhibitor mix (Roche), 1 mM EDTA). The soluble fraction was collected by centrifugation at 16,000 g for 10 minutes at 4°C on a benchtop centrifuge. Protein concentration was then determined by the DC Protein Assay (Bio-Rad) and samples were normalized to 2 µg/mL in native lysis buffer and 50 µL aliquots were distributed into PCR strip tubes. Samples were heated using a CETSA PCR Program (25°C, 3:00; 30–50°C gradient, 10:00; 25°C, 1:00) on a thermocycler. The soluble fraction was once again collected by removing the supernatant following centrifugation at 16,000 g for 10 minutes at 4°C. Prior to resolving equal volumes by SDS-PAGE, one third the volume of 3X SDS buffer was added to each sample. Membranes were immunoblotted with a mouse anti-His primary antibody (Ablab, 1:2,500) and a secondary antibody (Mandel Scientific, 1:10,000).

### Solubility assay

Cells expressing Guk1-7-GFP in log phase were incubated at either 25°C or 37°C for 20 minutes. Cells were lysed with glass beads in native lysis buffer (20 mM HEPES, pH 7.5, 0.5% NP-40, 200 mM NaCl, 1X protease inhibitor mix (Roche), 1 mM 1,10 phenanthroline, 1 mM EDTA) and centrifuged at 2,000 g for 5 minutes at 4°C. Protein concentrations were determined using the DC Protein Assay (Bio-Rad) and normalized to 0.5 µg/µL. Samples were then fractionated into soluble and pellet fractions by centrifuging at 16,000 g for 10 minutes at 4°C. The pellet fraction was washed twice with native lysis buffer prior to being resuspended in 1X SDS buffer (50 mM Tris-HCl, pH 6.8, 2% SDS, 3% glycerol). Equal volumes of total cell lysate, soluble, and pellet fractions were resolved by SDS-PAGE. Membranes were immunoblotted with mouse anti-GFP (Roche, 1:2,500) and secondary antibodies (Mandel Scientific, 1:10,000).

### Deg1 turnover assay

Cells transformed with a Deg1-GFP containing plasmid were grown to saturation overnight at 30°C, diluted to OD_600_ = 0.2 and then incubated for 3 hours at 30°C. Deg1-GFP expression was induced for 4 hours at 30°C with 100 µM copper sulphate and then 100 µg/mL cycloheximide was added with samples collected at the indicated time points. Cells were lysed with glass beads in lysis buffer (50 mM Tris-HCl, pH 7.5, 1% Tx-100, 0.1% SDS, 150 mM NaCl, 5 mM EDTA, 1 mM PMSF, 1X protease inhibitor mix (Roche)). Protein concentrations were measured using the DC Protein Assay (Bio-Rad) and normalized prior to resolving equal volumes by SDS-PAGE. Membranes were immunoblotted with mouse anti-GFP (Roche, 1:2,500) and rabbit anti-Pgk1 (Acris Antibodies, 1:10,000) primary antibodies and secondary antibodies (Mandel Scientific, 1:10,000). Membranes were scanned and analyzed with an Odyssey infrared imaging system (LI-COR).

### Guk1-7-GFP ubiquitination

Cells expressing ectopic Guk1-7-GFP, Guk1-GFP, or a control empty vector (pRS313) were grown to log phase and then lysed with glass beads in native lysis buffer (20 mM HEPES, pH 7.5, 0.5% NP-40, 200 mM NaCl, 1X protease inhibitor mix (Roche), 1 mM 1,10 phenanthroline, 1 mM EDTA, 10 mM iodoacetamide). GFP-tagged Guk1-7 was pulled down with GFP-Trap coupled agarose beads (Chromotek; 10 µL per 3 mg of lysate) for 2 hours at 4°C. Beads were washed three times in lysis buffer before samples were eluted with 3X SDS buffer. Equal volumes of samples were resolved by SDS-PAGE. Membranes were immunoblotted with mouse anti-GFP (Roche, 1:2,500), rabbit anti-Pgk1 (Acris Antibodies, 1:10,000), and mouse anti-ubiquitin (Millipore, 1:2,500) primary antibodies and secondary antibodies (Mandel Scientific, 1:10,000).

### Fluorescence microscopy

Cultures grown to log phase were divided and then incubated at either 25°C or 37°C for 20 minutes and then fixed in 3.7% formaldehyde for 15 minutes at room temperature. Fixed cells were rinsed with 1X PPS (0.1 M potassium phosphate, pH 7.5 with 1 M sorbitol) and then permeabilized with 0.1% Triton X-100 in 1X PPS for 10 minutes. For nuclear staining, permeabilized cells were incubated for 10 minutes with 1 µg/mL Hoechst 33342 in 1X PPS. Slides were prepared by mounting cells in 1X PPS and covered using concanavalin A treated coverslips. Imaging was performed with a 63X oil immersion objective on an inverted Zeiss Axio Observer microscope. Global contrast was adjusted using Zeiss Zen Pro software and images were then cropped for presentation in ImageJ.

### Statistical analysis

Data are presented as mean ± SD unless otherwise stated. Comparisons were made using the two-tailed Student’s *t*-test and differences were considered significant at a p-value of <0.05. When indicated, multiple strains were compared with a one-way ANOVA and post-hoc Tukey HSD to assess significance.

## Electronic supplementary material

Supplementarty Information
