# Supplementary material for: Recurrent background mutations in WHI2 impair proteostasis and degradation of misfolded cytosolic proteins in Saccharomyces cerevisiae
Source: Sci Rep. 2017 Jun 23;7:4183. doi: 10.1038/s41598-017-04525-8 (PMC5482819; doi:10.1038/s41598-017-04525-8)
Supplement: Supplementary file 1 — Supplementarty Information [file 41598_2017_4525_MOESM1_ESM.pdf]

Supplementary Information

Recurrent background mutations in *WHI2* impair proteostasis and degradation of misfolded cytosolic proteins in *Saccharomyces cerevisiae*

Sophie A. Comyn[1,2], Stéphane Flibotte[3], Thibault Mayor[2,*]

Supplementary Table 1. E3 ligase deletion strains used in screening

Systematic Name	Standard Name	Well No.	Systematic Name	Standard Name	Well No.	Systematic Name	Standard Name	Well No.
YMR258C	ROY1	A1	YIL030C	DOA10	C1	YJL157C	FAR1	E1
YOL013C	HRD1	A2	YBR114W	RAD16	C2	YDL013W	SLX5	E2
YOL054W	PSH1	A3	YDR457W	TOM1	C3	YBR203W	COS111	E3
YML068W	ITT1	A4	YGL141W	HUL5	C4	YKL059C	MPE1	E4
YDR049W	VMS1	A5	YGR184C	UBR1	C5	YER068W	NOT4	E5
YDR131C	n/a	A6	YDL074C	BRE1	C6	YMR026C	PEX12	E6
YDR143C	SAN1	A7	YLR224W	n/a	C7	YJL210W	PEX2	E7
YHR115C	DMA1	A8	YLR097C	HRT3	C8	YOR191W	ULS1	E8
YKL010C	UFD4	A9	YNL311C	SKP2	C9	YDR255C	RMD5	E9
YKL034W	TUL1	A10	YDR219C	MFB1	C10	YGL131C	SNT2	E10
YJL149W	DAS1	A11	YLR427W	MAG2	C11	YLR005W	SSL1	E11
YLR247C	IRC20	A12	YOR080W	DIA2	C12	YDR103W	STE5	E12
YNL230C	ELA1	B1	YHL010C	ETP1	D1	YDR266C	HEL2	F1
YKR017C	HEL1	B2	YLR368W	MDM30	D2	YOL138C	RTC1	F2
YDR265W	PEX10	B3	YER116C	SLX8	D3	YBR158W	AMN1	F3
YDR306C	n/a	B4	YLR352W	n/a	D4	YJR052W	RAD7	F4
YDR313C	PIB1	B5	YAL002W	VPS8	D5	YDR132C	n/a	F5
YIL001W	n/a	B6	YDR360W	TFB3	D6	YLR108C	n/a	F6
YCR066W	RAD18	B7	YLR024C	UBR2	D7	YMR080C	NAM7	F7
YJR036C	HUL4	B8	YMR119W	ASI1	D8	YPL046C	ELC1	F8
YDL190C	UFD2	B9	YNL008C	ASI3	D9	YJR090C	GRR1	F9
YBR062C	n/a	B10	YGL003C	CDH1	D10	YJL204C	RCY1	F10
YNL116W	DMA2	B11	YMR247C	RKR1	D11			
YBR280C	SAF1	B12	YNL023C	FAP1	D12			

Supplementary Table 2. Yeast strains used in this study.

**Strain ID**	**Alias**	**Genotype**	**Source**
YTM 408	BY4741	*MATa his3Δ1, leu2Δ0, ura3Δ0, met15Δ0*	Open Biosystems Collection
YTM 409	BY4742	*MATa his3Δ1, leu2Δ0, ura3Δ0, lys2Δ0*	Open Biosystems Collection
YTM 1690	*glo4Δ*	*MATa his3Δ1, leu2Δ0, ura3Δ0, met15Δ0, glo4Δ::KanMX4*	Open Biosystems Collection
YTM 1691	*whi2Δ*	*MATa his3Δ1, leu2Δ0, ura3Δ0, met15Δ0, whi2Δ::KanMX4*	Open Biosystems Collection
YTM 1744	*msn2Δ*	*MATa his3Δ1, leu2Δ0, ura3Δ0, met15Δ0, msn2Δ::KanMX4*	Open Biosystems Collection
YTM 1745	*msn4Δ*	*MATa his3Δ1, leu2Δ0, ura3Δ0, met15Δ0, msn4Δ::KanMX4*	Open Biosystems Collection
YTM 1856	ASI1 Spore 4c	*MATa his3Δ1, leu2Δ0, ura3Δ0, MET15, lys2Δ0, WHI2*	This paper
YTM 1857	ASI1 Spore 1a	*MATa his3Δ1, leu2Δ0, ura3Δ0, MET15, lys2Δ0, whi2-sc1*	This paper
YTM 1860	*asi1Δ*	*MATa his3Δ1, leu2Δ0, ura3Δ0, met15Δ0, asi1Δ::KanMX4, whi2-sc1*	Open Biosystems Collection
YTM 1861	*das1Δ*	*MATa his3Δ1, leu2Δ0, ura3Δ0, met15Δ0, das1Δ::KanMX4, whi2-2*	Open Biosystems Collection
YTM 1862	*fap1Δ*	*MATa his3Δ1, leu2Δ0, ura3Δ0, met15Δ0, fap1Δ::KanMX4, whi2-3*	Open Biosystems Collection
YTM 1863	*hrt3Δ*	*MATa his3Δ1, leu2Δ0, ura3Δ0, met15Δ0, hrt3Δ::KanMX4, whi2-4*	Open Biosystems Collection
YTM 1864	*hul5Δ*	*MATa his3Δ1, leu2Δ0, ura3Δ0, met15Δ0, hul5Δ::KanMX4, whi2-5*	Open Biosystems Collection
YTM 1865	*ufd2Δ*	*MATa his3Δ1, leu2Δ0, ura3Δ0, met15Δ0, ufd2Δ::KanMX4, whi2-6*	Open Biosystems Collection
YTM 1866	*ufd4Δ*	*MATa his3Δ1, leu2Δ0, ura3Δ0, met15Δ0, ufd4Δ::KanMX4, whi2-7*	Open Biosystems Collection

Strain ID	Alias	Genotype	Source
YTM 1867	*asi1*Δ Spore 3a	*MATa his3Δ1, leu2Δ0, ura3Δ0, MET15, LYS2, asi1Δ::KanMX4, whi2-1*	This paper
YTM 1868	*asi1*Δ Spore 3b	*MATa his3Δ1, leu2Δ0, ura3Δ0, met15Δ0, lys2Δ0, asi1Δ::KanMX4, WHI2*	This paper
YTM 1869	ASI1 Spore 3c	*MATa his3Δ1, leu2Δ0, ura3Δ0, MET15, LYS2, whi2-1*	This paper
YTM 1870	ASI1 Spore 3d	*MATa his3Δ1, leu2Δ0, ura3Δ0, met15Δ0, lys2Δ0, WHI2*	This paper
YTM 1871	*asi1*Δ Spore 3a / BY4741	*MATa/MATa his3Δ1/his3Δ1, leu2Δ0/leu2Δ0, ura3Δ0/ura3Δ0, met15Δ0/MET15, LYS2/lys2Δ0, asi1Δ::KanMX4/ASI1, whi2-1/WHI2*	This paper
YTM 1872	*asi1*Δ Spore 3a / *asi1*Δ	*MATa/MATa his3Δ1/his3Δ1, leu2Δ0/leu2Δ0, ura3Δ0/ura3Δ0, met15Δ0/MET15, LYS2/lys2Δ0, asi1Δ::KanMX4/asi1Δ::KanMX4, whi2-1/whi2-1*	This paper
YTM 1873	*asi1*Δ Spore 3a / *das1*Δ	*MATa/MATa his3Δ1/his3Δ1, leu2Δ0/leu2Δ0, ura3Δ0/ura3Δ0, met15Δ0/MET15, LYS2/lys2Δ0, asi1Δ::KanMX4/ASI1, DAS1/das1Δ::KanMX4, whi2-1/whi2-2*	This paper
YTM 1874	*asi1*Δ Spore 3a / *fap1*Δ	*MATa/MATa his3Δ1/his3Δ1, leu2Δ0/leu2Δ0, ura3Δ0/ura3Δ0, met15Δ0/MET15, LYS2/lys2Δ0, asi1Δ::KanMX4/ASI1, FAP1/fap1Δ::KanMX4, whi2-1/whi2-3*	This paper
YTM 1875	*asi1*Δ Spore 3a / *hrt3*Δ	*MATa/MATa his3Δ1/his3Δ1, leu2Δ0/leu2Δ0, ura3Δ0/ura3Δ0, met15Δ0/MET15, LYS2/lys2Δ0, asi1Δ::KanMX4/ASI1, HRT3/hrt3Δ::KanMX4, whi2-1/whi2-4*	This paper
YTM 1876	*asi1*Δ Spore 3a / *hul5*Δ	*MATa/MATa his3Δ1/his3Δ1, leu2Δ0/leu2Δ0, ura3Δ0/ura3Δ0, met15Δ0/MET15, LYS2/lys2Δ0, asi1Δ::KanMX4/ASI1, HUL5/hul5Δ::KanMX4, whi2-1/whi2-5*	This paper
YTM 1877	*asi1*Δ Spore 3a / *ufd2*Δ	*MATa/MATa his3Δ1/his3Δ1, leu2Δ0/leu2Δ0, ura3Δ0/ura3Δ0, met15Δ0/MET15, LYS2/lys2Δ0, asi1Δ::KanMX4/ASI1, UFD2/ufd2Δ::KanMX4, whi2-1/whi2-6*	This paper
YTM 1878	*asi1*Δ Spore 3a / *ufd4*Δ	*MATa/MATa his3Δ1/his3Δ1, leu2Δ0/leu2Δ0, ura3Δ0/ura3Δ0, met15Δ0/MET15, LYS2/lys2Δ0, asi1Δ::KanMX4/ASI1, UFD4/ufd4Δ::KanMX4, whi2/whi2-7*	This paper
YTM 1879	*asi1*Δ Spore 3b / BY4741	*MATa/MATa his3Δ1/his3Δ1, leu2Δ0/leu2Δ0, ura3Δ0/ura3Δ0, met15Δ0/MET15, LYS2/lys2Δ0, asi1Δ::KanMX4/ASI1, WHI2/WHI2*	This paper
YTM 1880	*asi1*Δ Spore 3b / *asi1*Δ	*MATa/MATa his3Δ1/his3Δ1, leu2Δ0/leu2Δ0, ura3Δ0/ura3Δ0, met15Δ0/MET15, LYS2/lys2Δ0, asi1Δ::KanMX4/asi1Δ::KanMX4, WHI2/whi2-1*	This paper
YTM 1881	*asi1*Δ Spore 3b / *das1*Δ	*MATa/MATa his3Δ1/his3Δ1, leu2Δ0/leu2Δ0, ura3Δ0/ura3Δ0, met15Δ0/MET15, LYS2/lys2Δ0, asi1Δ::KanMX4/ASI1, DAS1/das1Δ::KanMX4, WHI2/whi2-2*	This paper
YTM 1882	*asi1*Δ Spore 3b / *fap1*Δ	*MATa/MATa his3Δ1/his3Δ1, leu2Δ0/leu2Δ0, ura3Δ0/ura3Δ0, met15Δ0/MET15, LYS2/lys2Δ0, asi1Δ::KanMX4/ASI1, FAP1/fap1Δ::KanMX4, WHI2/whi2-3*	This paper
YTM 1883	*asi1*Δ Spore 3b / *hrt3*Δ	*MATa/MATa his3Δ1/his3Δ1, leu2Δ0/leu2Δ0, ura3Δ0/ura3Δ0, met15Δ0/MET15, LYS2/lys2Δ0, asi1Δ::KanMX4/ASI1, HRT3/hrt3Δ::KanMX4, WHI2/whi2-4*	This paper

**Strain ID**	**Alias**	**Genotype**	**Source**
YTM 1884	*asi1*Δ Spore 3b / *hul5*Δ	*MATa/MATa his3Δ1/his3Δ1, leu2Δ0/leu2Δ0, ura3Δ0/ura3Δ0, met15Δ0/MET15, LYS2/lys2Δ0, asi1Δ::KanMX4/ASI1, HUL5/hul5Δ::KanMX4, WHI2/whi2-5*	This paper
YTM 1885	*asi1*Δ Spore 3b / *ufd2*Δ	*MATa/MATa his3Δ1/his3Δ1, leu2Δ0/leu2Δ0, ura3Δ0/ura3Δ0, met15Δ0/MET15, LYS2/lys2Δ0, asi1Δ::KanMX4/ASI1, UFD2/ufd2Δ::KanMX4, WHI2/whi2-6*	This paper
YTM 1886	*asi1*Δ Spore 3b / *ufd4*Δ	*MATa/MATa his3Δ1/his3Δ1, leu2Δ0/leu2Δ0, ura3Δ0/ura3Δ0, met15Δ0/MET15, LYS2/lys2Δ0, asi1Δ::KanMX4/ASI1, UFD4/ufd4Δ::KanMX4, WHI2/whi2-7*	This paper
YTM1980	Hsp12-GFP, *whi2-sc1*	*MATa his3Δ1, leu2Δ0, ura3Δ0, MET15, LYS2, whi2-1, Hsp12-GFP-His3MX6*	This paper
YTM1981	Hsp12-GFP, *WHI2*	*MATa his3Δ1, leu2Δ0, ura3Δ0, met15Δ0, lys2Δ0, WHI2, Hsp12-GFP-His3MX6*	This paper
YTM1984	Hsp42-GFP, *whi2-sc1*	*MATa his3Δ1, leu2Δ0, ura3Δ0, MET15, LYS2, whi2-1, Hsp42-GFP-His3MX6*	This paper
YTM1985	Hsp104-GFP, *whi2-sc1*	*MATa his3Δ1, leu2Δ0, ura3Δ0, MET15, LYS2, whi2-1, Hsp104-GFP-His3MX6*	This paper
YTM1986	Hsp104-GFP, *WHI2*	*MATa his3Δ1, leu2Δ0, ura3Δ0, met15Δ0, lys2Δ0, WHI2, Hsp104-GFP-His3MX6*	This paper
YTM1987	Hsp42-GFP, *WHI2*	*MATa his3Δ1, leu2Δ0, ura3Δ0, met15Δ0, lys2Δ0, WHI2, Hsp42-GFP-His3MX6*	This paper
YTM2020	*msn2*Δ*whi2*Δ	*MATa his3Δ1, leu2Δ0, ura3Δ0, met15Δ0, msn2Δ::KanMX4, whi2Δ::NatMX*	This paper

Supplementary Table 3. Plasmids used in this study.

Plasmid ID	Name	Auxotrophic Marker	Genotype	Source
BPM 42	pRS316	Ura	CEN/ARS	RJD Collection
BPM 45	pRS313	His	CEN/ARS	RJD Collection
BPM 49	pRS315	Leu	CEN/ARS	RJD Collection
BPM 453	P_{GPD}-Guk1-GFP	His	CEN/ARS	T. Mayor
BPM 458	P_{GPD}-Guk1-7-GFP	His	CEN/ARS	T. Mayor
BPM 609	P_{GPD}-Guk1-7-GFP	Leu	CEN/ARS	T. Mayor
BPM 708	P_{CUP1}-Deg1-GFP	Ura	CEN/ARS	T. Mayor
BPM 718	P_{GPD}-Guk1-7-GFP	Ura	CEN/ARS	T. Mayor
BPM 748	P_{ASI1}-ASI1	Leu	CEN/ARS	This paper
BPM 749	P_{DAS1}-DAS1	Leu	CEN/ARS	This paper
BPM 750	P_{FAP1}-FAP1	Leu	CEN/ARS	This paper
BPM 751	P_{HRT3}-HRT3	Leu	CEN/ARS	This paper
BPM 752	P_{HUL5}-HUL5	Leu	CEN/ARS	This paper
BPM 753	P_{UFD2}-UFD2	Leu	CEN/ARS	This paper
BPM 754	P_{UFD4}-UFD4	Leu	CEN/ARS	This paper
BPM 863	P_{WHI2}-WHI2	Leu	CEN/ARS	This paper
BPM 914	P_{WHI2}-WHI2	Ura	CEN/ARS	This paper

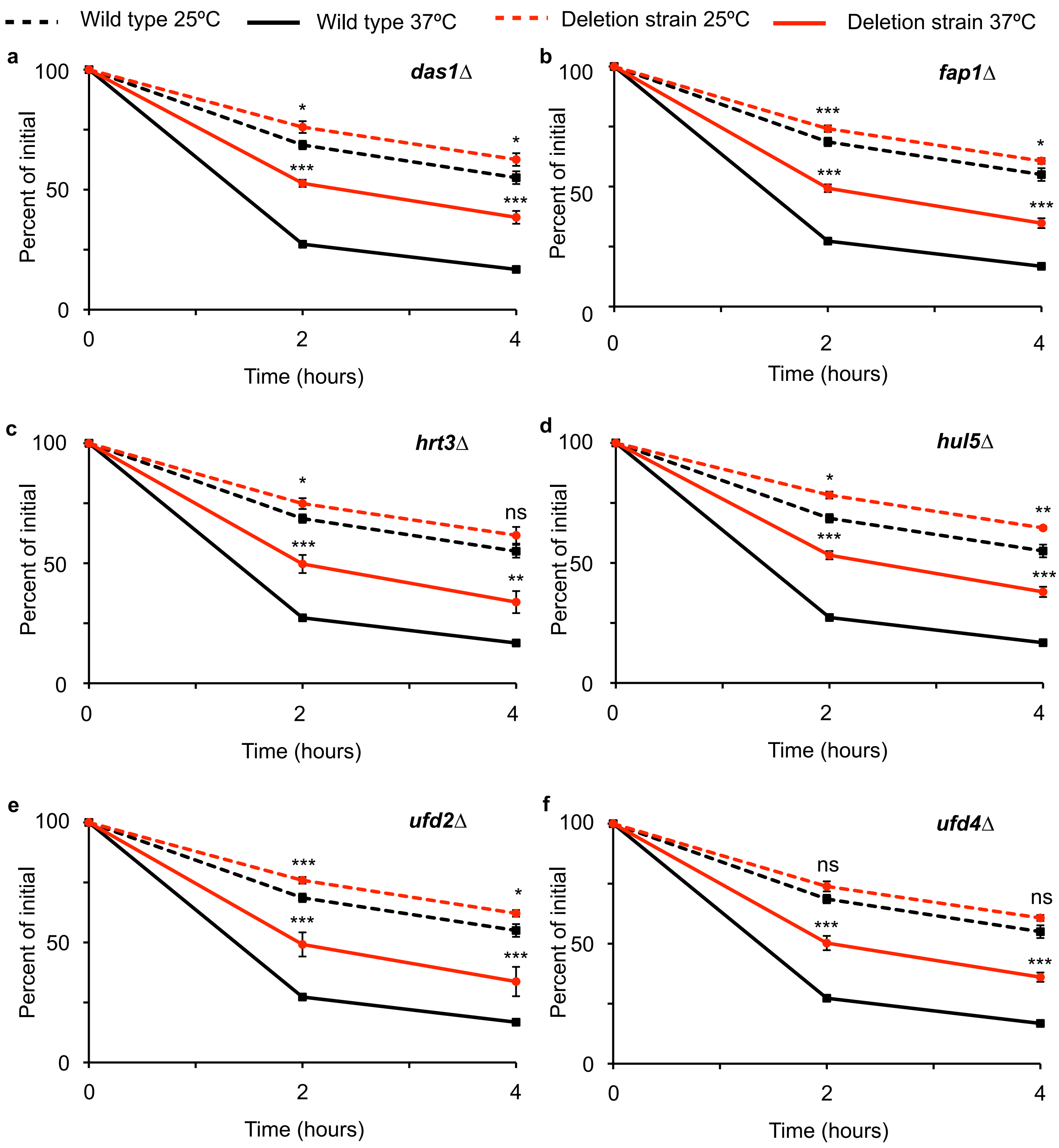

Supplementary Figure 1. Guk1-7-GFP degradation in E3 ligase deletion strains. CHX chase assay. Wild type or the designated E3 ligase deletion strains expressing ectopic Guk1-7-GFP were incubated in the presence of CHX at either 25ºC or 37ºC for four hours and samples were analysed by flow cytometry at the indicated time points. Results represent the mean and standard deviation of three independent experiments. P values were calculated with a two-tailed unpaired Student's *t*-test (*, **, ***, and ns denote $p < 0.05, 0.01, 0.005$, and not significant, respectively). a) *das1*Δ*,* b) *fap1*Δ, c) *hrt3*Δ, d) *hul5*Δ, e) *ufd2*Δ, f) *ufd4*Δ.

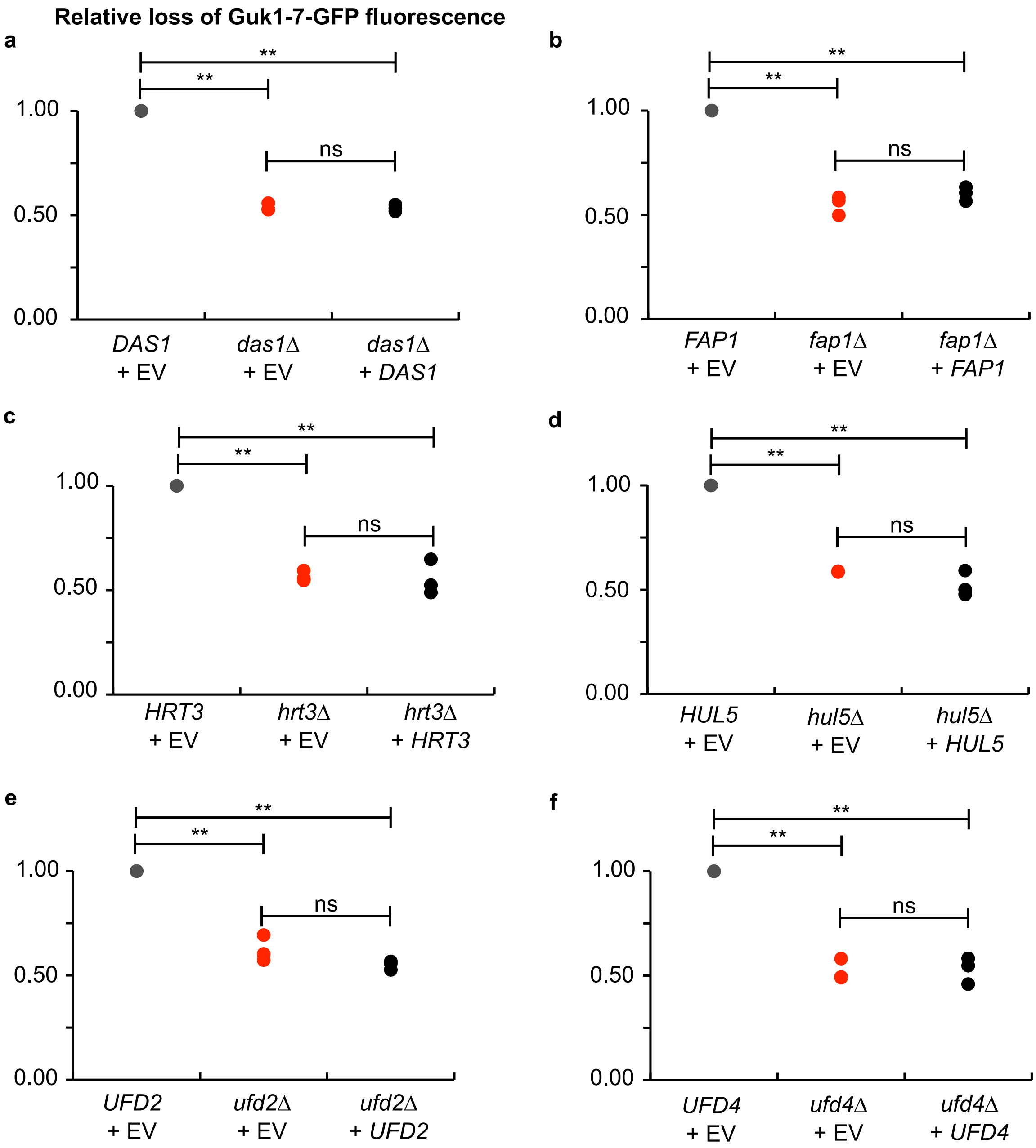

Supplementary Figure 2. Guk1-7-GFP stability is not a direct effect of E3 ligase deletion. Wild type and E3 ligase deletion strains expressing Guk1-7-GFP along with an empty vector (EV) control or corresponding E3 gene under its endogenous promoter and terminator were incubated with CHX at 25ºC or 37ºC for two hours prior to flow cytometry analysis. Results represent three independent experiments. P values were calculated with a one-way ANOVA and post-hoc Tukey HSD to assess significance (** and ns denotes $p < 0.01$ and not significant, respectively). a) *das1*Δ b) *fap1*Δ c) *hrt3*Δ d) *hul5*Δ e) *ufd2*Δ f) *ufd4*Δ.

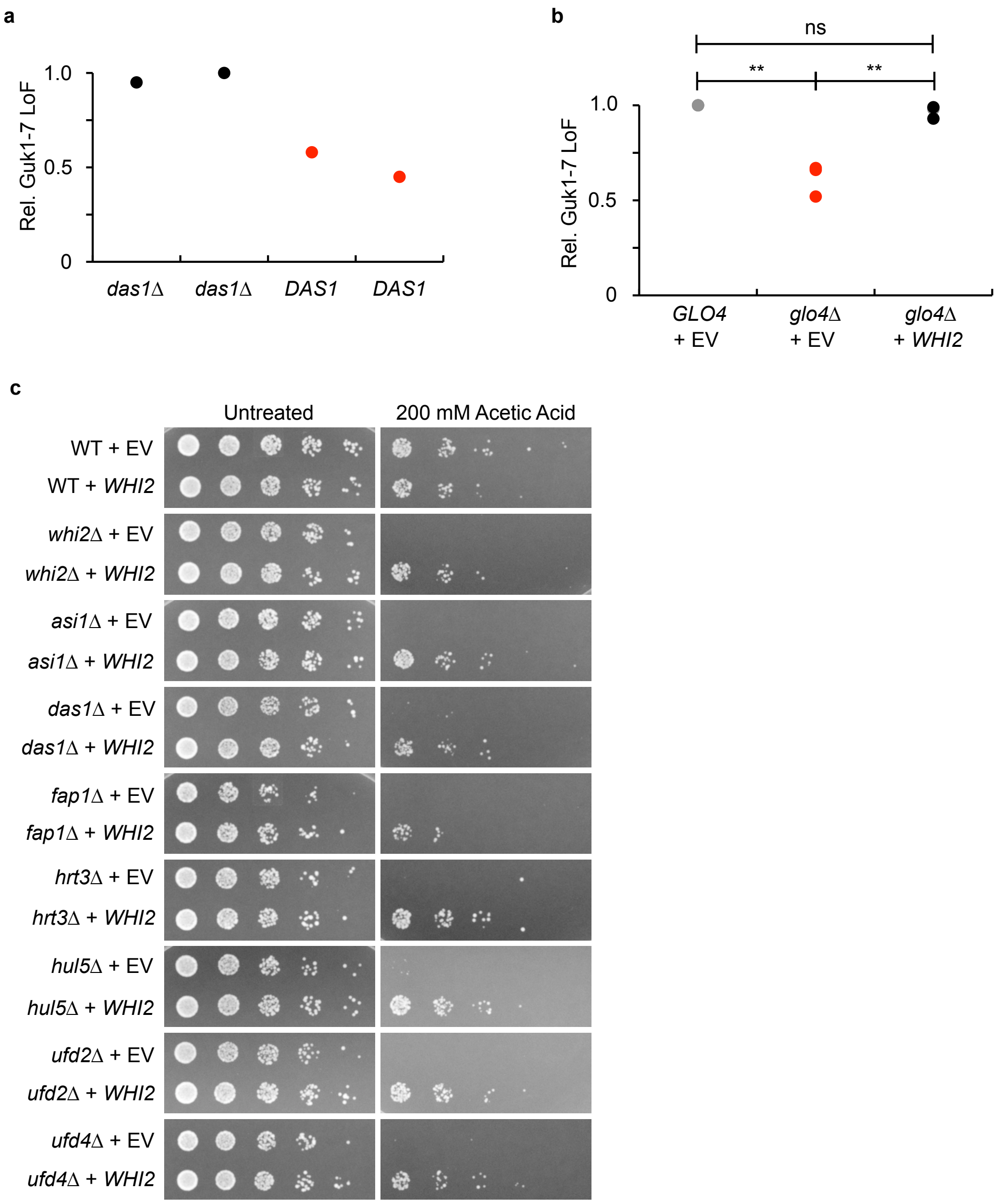

Supplementary Figure 3. *das1*Δ tetrad analysis and *WHI2* addback. a) Analysis of one tetrad obtained from backcrossing the *MAT*a *das1*Δ strain with the wild type *MAT*alpha BY4742. Tetrad spores expressing Guk1-7-GFP were incubated with CHX at 25ºC or 37ºC for two hours prior to flow cytometry analysis. b) Wild type and *glo4*Δ cells co-expressing Guk1-7-GFP and an empty control vector (EV) or *WHI2* were incubated with CHX at 25ºC or 37ºC for two hours and then analysed by flow cytometry. Results represent three independent experiments and p values were calculated with a one-way ANOVA and post-hoc Tukey HSD to assess significance (** and ns denote $p < 0.01$ and not significant, respectively). c) Diluted overnight cultures of wild type or E3 ligase deletion strains expressing either an empty control vector or *WHI2* were treated with 200 mM acetic acid for four hours prior to serial dilution and spotting onto synthetic drop out plates. Images were taken after two days of growth at 30ºC.

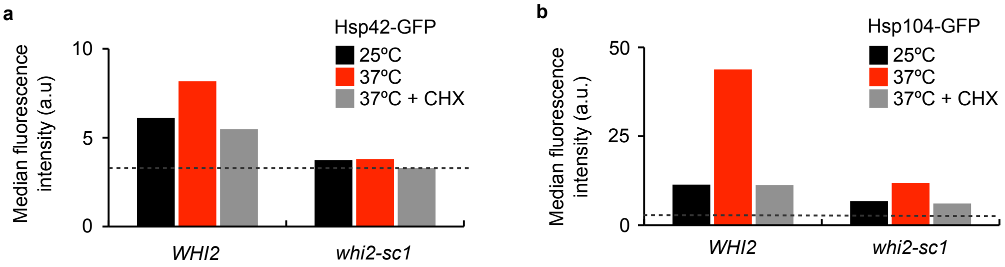

Supplementary Figure 4. Msn2 target protein levels are decreased in *whi2-sc1* cells. *WHI2* and *whi2-sc1* cells with Hsp42 (a) or Hsp104 (b) endogenously tagged with GFP were grown to log phase and then incubated at 25ºC, 37ºC, or 37ºC in the presence of CHX for two hours prior to analysis by flow cytometry. The horizontal dotted line represents the median background fluorescence.

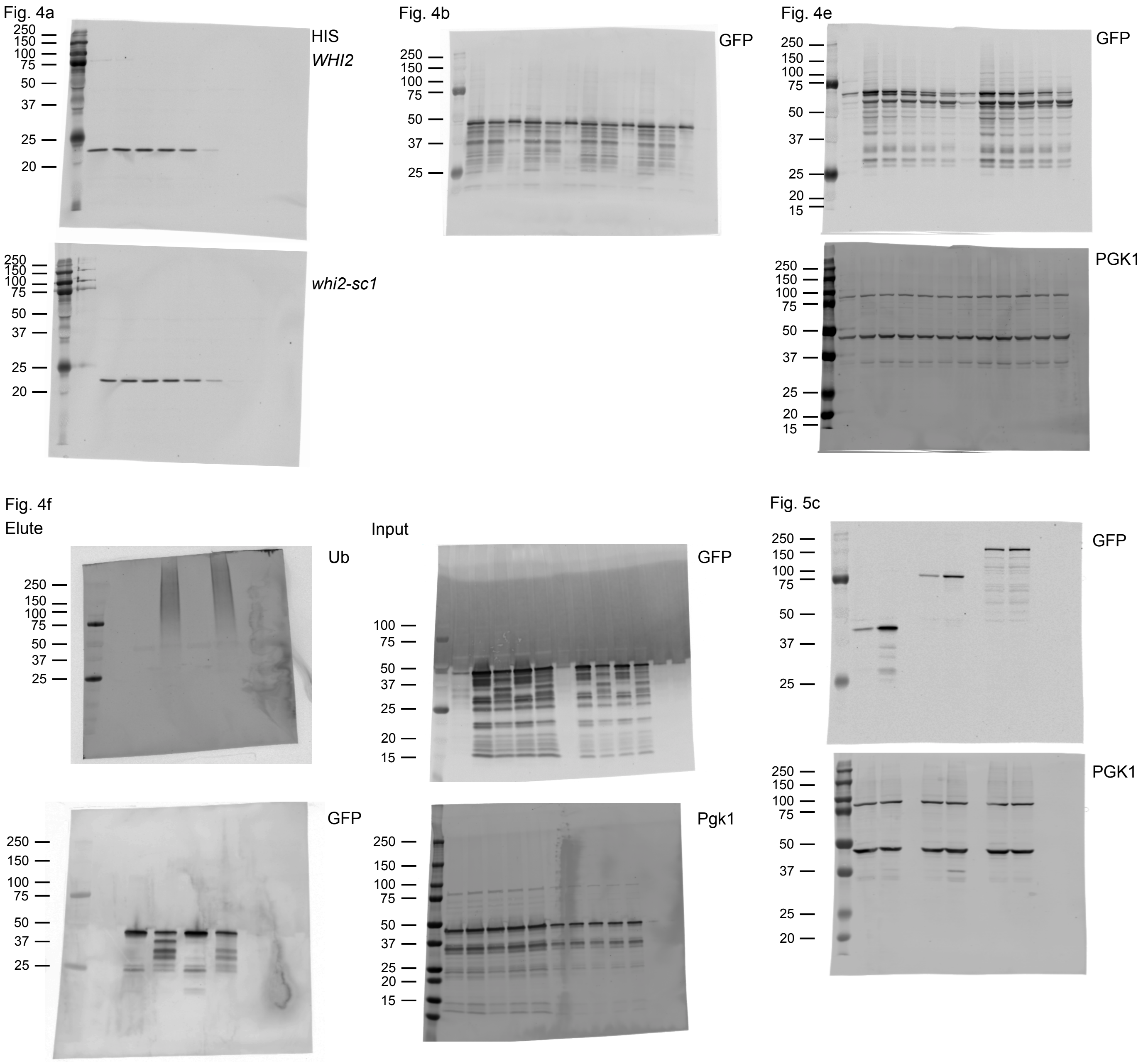

Supplementary Figure 5. Uncropped images for all Western blots displayed in main figures.
